# Autism, menstruation and mental health- a scoping review and a call to action

**DOI:** 10.3389/fgwh.2025.1531934

**Published:** 2025-06-25

**Authors:** Joanna Skommer, Krish Gunesh

**Affiliations:** ^1^ Department of Psychiatry, Central Adelaide Health Network, SA Health, Adelaide, SA, Australia; ^2^ Department of Psychiatry, Central Metropolitan CAMHS Western, Women’s and Children Health Network, Adelaide, SA, Australia

**Keywords:** autism spectrum disorder, autistic individuals, menstruation, menarche, menopause, sexspecific medicine

## Abstract

**Introduction:**

There is increasing evidence regarding the mental health implications of cyclical hormonal fluctuations associated with menstruation, as well as of key reproductive transitions (menarche and menopause), in typically developing individuals. Autism spectrum disorder (ASD), a complex neurodevelopmental condition, may predispose individuals to maladaptive responses to life changes such as menstruation. Despite the importance of this topic, research relating to menstrual experiences across the lifespan of autistic adults remains scarce, largely due to the intersecting effects of multiple marginalizing characteristics experienced by this population. This research gap significantly limits our understanding of how menstruation impacts the mental health of autistic individuals.

**Objectives:**

The purpose of this scoping review was to examine existing evidence about menstrual experiences, including menarche and menopause, and their impact on mental health among autistic individuals, and propose a biopsychosocial framework for the complex interplay of individual, healthcare, and societal vulnerabilities that predispose autistic individuals to negative menstrual experiences.

**Methods:**

A scoping review of original articles, quantitative and qualitative, published in English from 1980 onwards, identified through search of online databases and reference lists, using PRISMA extension for scoping reviews.

**Results:**

A total of 45 studies were identified to meet the specified inclusion and exclusion criteria. The key emerging themes were the mental health impact of menstruation, the occurrence and experience of menstrual disorders among autistic individuals, as well as support strategies and healthcare utilization by that population.

**Conclusions:**

Although our current knowledge on menstrual health specific to autistic individuals is still scant, it nevertheless raises significant concerns regarding potential challenges. The findings of this study have been placed within the bio-psycho-socio-cultural framework to emphasize that menstrual experiences occur within the context of person-environment transactions, and that autistic individuals are vulnerable to negative menstrual experiences because of adverse or non-facilitative societal and healthcare environments. Further large-scale studies addressing identified gaps (e.g., influence of gender diversity, impact of medical comorbidities, trauma and stigma) is warranted.

**Systematic Review Registration:**

https://osf.io/gxurq.

## Introduction

The menstrual cycle is characterized by recurrent fluctuations in estrogen and progesterone levels, which can influence mental health via several different mechanisms. Physical discomfort during menstruation can correlate with psychological distress and irritability, and reduced self-esteem (1). Additionally, many typically developing individuals indicate increased interpersonal conflicts and decline in social engagement before and during menstruation, potentially leading to feelings of isolation and a decrease in mood (2, 3). The menstrual cycle can also have direct biological impacts on mental health. Estrogen reduces dopamine transmission, mimicking antidopaminergic action of many antipsychotic medications (4). This may explain an increased vulnerability to psychosis when estrogen is low (e.g., during menstruation) (5). In the same vein, progesterone can have anxiolytic effect through increases in allopregnanolone and enhanced GABA potentiation (6–8). Unsurprisingly, studies in general population show that premenstrual and menstrual phases are implicated in transdiagnostic symptom exacerbation, including increases in psychosis, mania, depression, suicide attempts, and alcohol use (9–11). There is also increasing evidence that key reproductive transitions, such as menarche (the onset of menses) and menopause (the cessation of menses), have behavioural and psychosocial consequences in typically developing individuals (12–14). Considering the mental health implications of menstruation reported in typically developing individuals, it is interesting to examine how key reproductive transitions, and menstrual experiences, affect individuals with neurodevelopmental disorders, such as autism.

Autism spectrum disorder (ASD) is a complex neurodevelopmental condition characterized by deficits in reciprocal social communication, difficulty adapting to change, a repertoire of restrictive, repetitive, and stereotyped thought and behavioral patterns, as well as neurodivergence in sensorial perception (difference in sensory information processing involving both hypo- and hyperresponsiveness). Additionally, autistic individuals often experience co-occurring psychiatric disorders (15). Due to core features of ASD, autistic individuals often respond differently and at times maladaptively to novelty and changes in life patterns (16). As such, it can be postulated that autistic individuals may experience reproductive transitions and changes associated with menstruation differently – and more negatively – compared to typically developing individuals.Remarkably little is known, however, about the menstrual experiences of autistic individuals. This knowledge gap stems from several interconnected factors. First, the longstanding perspective of ASD as a developmental condition has directed most autism research and services towards children, neglecting the needs and experiences of autistic adults (17). Second, significant methodological challenges exist in conducting research involving individuals with neurodevelopmental disorders, including those with intellectual and/or communication disabilities. These challenges often lead to reliance on retrospective caregiver reports, which are prone to recall and rater bias, resulting in autistic individuals' needs being represented by proxies rather than through their own valuable perspectives and experiences. From a gender perspective, women have been consistently under-represented across research studies (18, 19), with clinical trials failing to adequately enroll women. Despite over half of the world's population experiencing menstruation, outcomes related to menstrual cycle are often an afterthought in clinical trials (19). The implications of hormonal fluctuations, whether endogenous or exogenous from contraceptives, remain underexplored, despite their potential to impact on mental health of menstruating individuals. For example, it is estimated that over 400 million females use hormonal contraceptives worldwide, and yet it was not until the 2016 Danish registry study that the mental health implications of hormonal contraception were analyzed in a large population (20). Autistic females have been further disproportionately excluded from research participation (21, 22), likely because of the field's historical androcentric stance - the clinical and societal-level perceptual biases viewing autism as a predominantly male disorder - and limited awareness of the female ASD phenotype (23). Highlighting this research gap, almost 80% of Autistic people who completed the AARC 2019 consultation on autism research priorities identified the need for research focused on women (24). Consequently, the paucity of research on menstrual health and experiences of autistic individuals across lifespan can be understood as resulting from the intersecting effects of multiple marginalizing characteristics.

The Australian “End Gender Bias” survey found that two-thirds of female patients reported experiencing gender bias or discrimination (25). This persistent stigmatization of female patients, combined with the underrepresentation of autistic individuals in menstrual health research, and a long-overlooked connection between reproductive and mental health, perpetuate a cascade of healthcare disparities that negatively impact health outcomes and healthcare quality (e.g., life expectancy, access to services) (26). Managing menstrual health with adequate knowledge, safety and dignity, without stigma and discrimination, is a fundamental human right that should not exclude neurodivergent individuals. By improving our understanding of how menstrual experiences affect mental health of autistic individuals, we have a valuable opportunity to improve patient care through highlighting potentially modifiable risk factors in clinical practice.

Therefore, a scoping review was conducted to examine existing evidence about menstrual experiences, including menarche and menopause, among autistic individuals, with emphasis on mental health impact (formal diagnoses and reported symptoms). It is important to note that autism itself is not considered a mental health condition, but rather a neurodevelopmental disorder. However, challenging behaviours commonly associated with ASD (e.g., aggression, self-injurious or compulsive behaviours, severe tantrumming), which can be classified as behavioural disorders, often fall under the purview of management by mental health clinicians and are thus also considered mental health issues. This comprehensive approach allowed us to gain a thorough understanding of the topic and identify gaps in current research. The themes we uncovered informed our development of a biopsychosocial perspective on the complex interplay of individual, healthcare, and societal vulnerabilities. This integrated perspective can guide future research directions, particularly regarding therapeutic management strategies for this population.

The language used in discussions about autism, gender, and menstrual health is highly debated (27, 28). We hence wish to highlight the terminology used here, namely “autistic individuals”. This identity-first language has been documented as the endorsed preference of a large percentage of autistic adults (27). Additionally, this phrasing aims to be inclusive, encompassing women, girls, transmen, and non-binary individuals. The term “menstruators” was avoided, as it reduces these individuals to their bodily functions or body parts.

## Aims

The current scoping review aims to map the research on menstruation experiences among autistic individuals, from menarche to menopause, and explore the unique challenges faced by this population, in comparison with the typically developing individuals, with particular emphasis on mental health, as well as identify gaps and opportunities for future research bridging the existing research areas.

## Materials and methods

### Approach

Scoping reviews help to produce comprehensive mappings of concepts, research topics and scientific evidence. The scoping review was performed using the PRISMA (Preferred Reporting Items for Systematic Reviews and Meta-Analyses) extension for scoping reviews (29) (Supplementary Table S1).

### Patient and public involvement

The study is a scoping review that does not involve patients or the public.

### Eligibility criteria

Publications were eligible for inclusion in this review if they were: (i) published in English; (ii) published after 1980 (the year of publication of DSM III); (iii) original papers (including letters to editor); (iv) either quantitative, qualitative studies or mixed-method studies; and (v) studies involving autistic individuals as defined by formal diagnosis, administrative records or by self-identification. The exclusion criteria: (i) studies not conducted on autistic population (e.g., other forms of neurodivergence, such as ADHD, or specific genetic syndromes); (ii) studies where menstruation was not treated as the concept in focus (e.g., pubertal timing without assessment of menstruation status or experience, menarche status mentioned only as a confounding variable; analysis of hormonal variables such as oxytocin rather than menstruation); (iii) studies on animal models of ASD; (iv) studies of maternal menstrual health rather than menstrual health of autistic individuals; (v) position statements/guides or study protocols rather than completed primary research.

### Search strategy

Search for peer-reviewed literature was conducted under the guidance of an experienced health Services librarian, initially between September and October 2024 with the final search in May 2025, in Medline/PubMED. The following search terms and their combinations were used: “autism”, OR “autism spectrum disorder”, AND either of the following terms: “menarche”, “menstruation, “menses”, “menopause”, dysmenorrhea”, “PMDD”, “premenstrual dysphoric disorder”, and “premenstrual” (Table 1). Date of publication limit (1980) was applied. No additional search limits were applied. The final search results were exported into Excel and duplicates were removed by the first author, with subsequent verification by the second author. Two authors (J.S. and K.G.) independently screened all titles and abstracts of potential references, excluding those that did not meet the search criteria. The full texts of the papers were also independently screened by both authors (J.S. and K.G.), with discrepancies resolved by consensus between the authors. Next, the two authors separately checked the included papers' reference lists for identifying additional relevant studies. In the final step, we glimpsed at Google scholar for articles citing current publications, maximizing our efforts to collect all relevant studies.

**Table 1:** Final PubMed queries.

Component	Term
Autism	1. Autism 2. Autism spectrum disorder
Menstruation	3. Menarche 4. Menstruation 5. Menses 6. Menopause 7. Dysmenorrhea 8. PMDD 9. Premenstrual dysphoric disorder 10. Premenstrual
Combination	11. 1 OR 2 12. 3 OR 4 OR 5 OR 6 OR 7 OR 8 OR 9 OR 10 13. 11 AND 12

**Figure 1:**
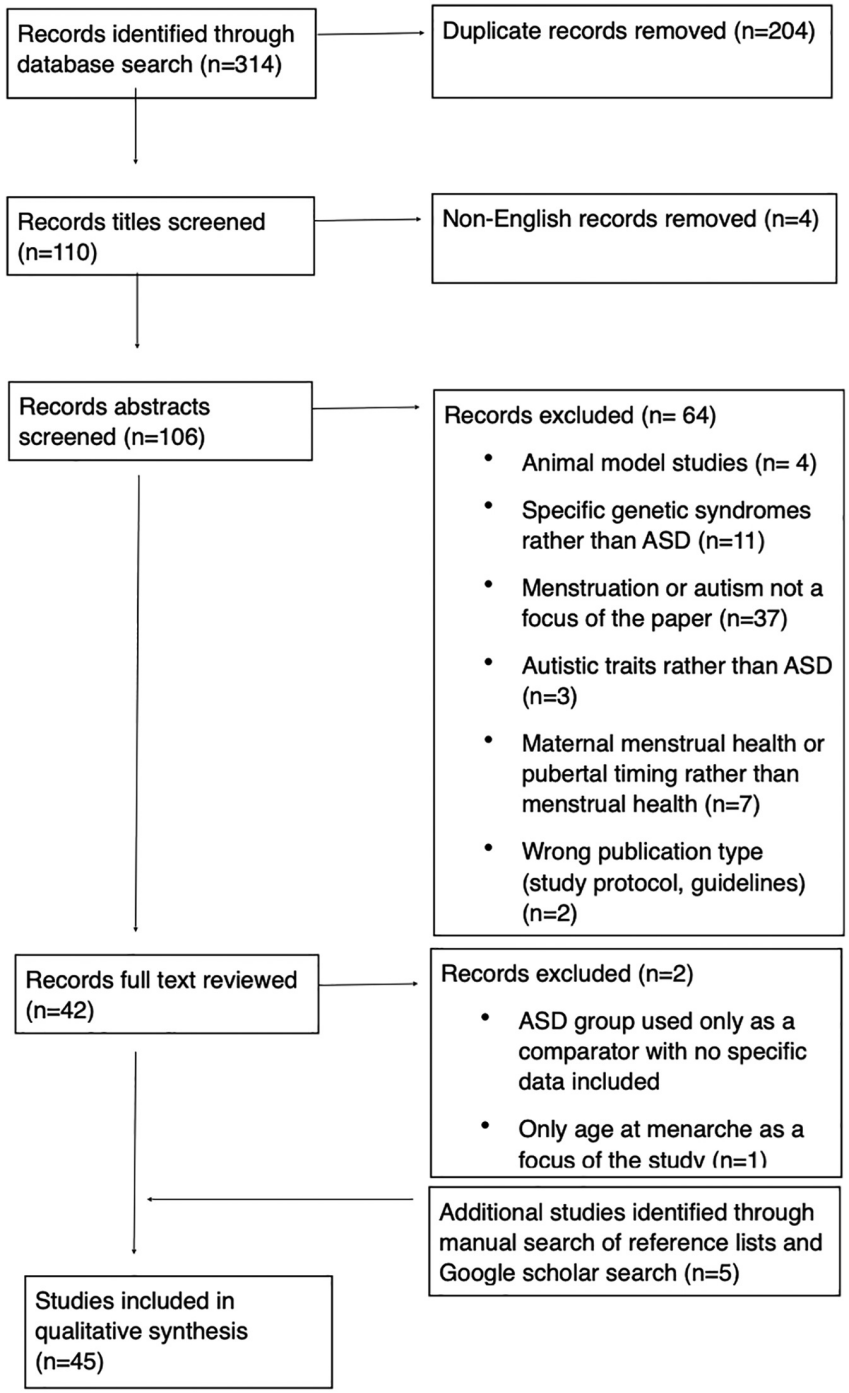
PRISMA flow diagram.

A total of 314 articles were found with the initial search. The total number of papers was reduced to 106 after removal of duplicates, and these were included in the initial screening by abstract and title whereby the papers that were not relevant were excluded. Full text of 40 papers was reviewed. The reference lists of these relevant articles (30–69) were hand-searched to identify any further studies, and google scholar searched for relevant articles citing current publications, and these were added manually (*n* = 5) (70–74). All steps are reported in the PRISMA diagram (Figure 1).

### Data charting and synthesis of results

A data-charting form was jointly developed by the authors to determine which variables to extract. Extracted data included specific details from the included studies: authors and year of publication, study location (country of origin), study design, aims of the studies (where applicable), study population characteristics (number and age), and summary of the results. Using the summary of the results, the charting tool was expanded to include identified themes under the three topic areas: (i) mental health, (ii) menstrual disorders, and (iii) menstruation management. Where we identified a systematic or scoping review, we analyzed the number of studies included in the review and noted if any studies had been missed by our search.

## Results

**Figure 2:**
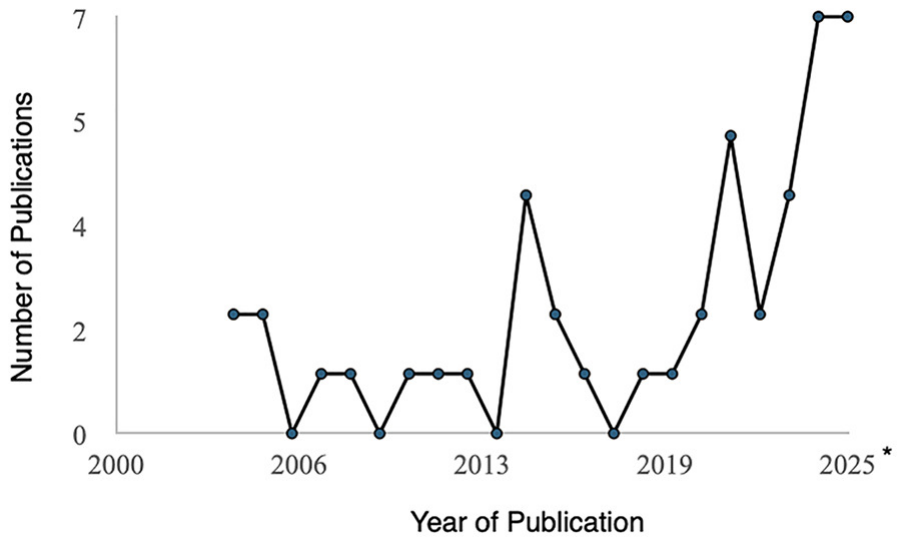
Number of studies published each year from the final studies included in this review. Only representative publications until 02 May 2025.

**Figure 3:**
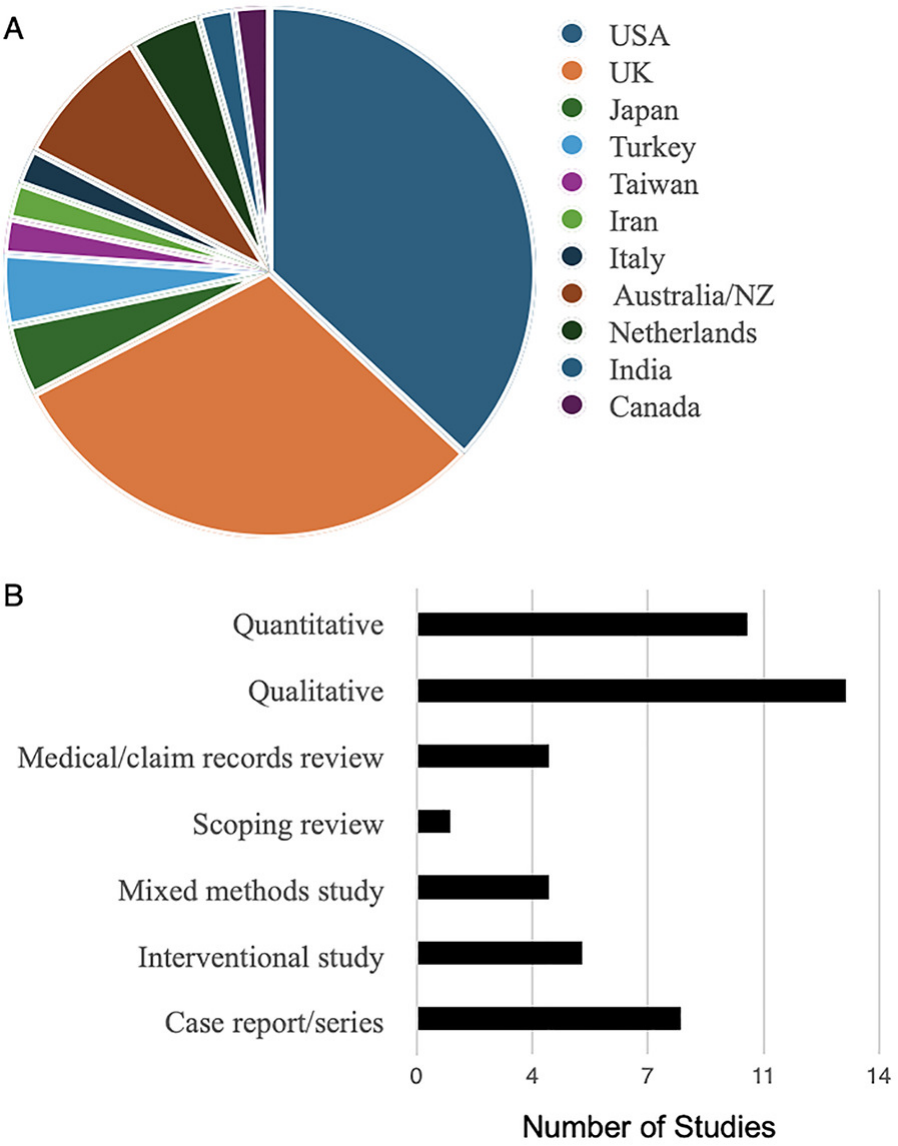
Study characteristics. **(A)** Country of origin for publications included in this review. NZ, New Zealand, UK – United Kingdom, USA – United States of America. **(B)** Study types.

The final 45 articles selected for this review were published between 2004 and 2025 (Figure 2). Interestingly, 27 out of 45 studies (60%) were published since 2020, and 15.5% in 2025 (as of May 2025), indicating that menstrual health research specific to autistic individuals has only recently received increased attention. In terms of the publication of origin, the studies originated mostly from USA and UK (68.9%; Figure 3A). The studies included a scoping review, eight case reports/series, four analyses of medical health records/medical claims, and five studies that used educational interventions (Supplementary Table S2; Figure 3B). There were thirteen qualitative studies, ten quantitative studies and four mixed-method studies (Figure 3B).

The key themes addressed in the reviewed studies were the mental health impact of menstruation, the occurrence and experience of menstrual disorders among autistic individuals, as well as support strategies and healthcare utilization by that population (Supplementary Table S2).

### Mental health impact of menstruation in autistic individuals

Twenty-six studies have examined the mental health impact of menstruation in autistic individuals, including affective and behavioral changes, as well as amplification of ASD symptoms such as behaviours of concern.

Research on menstruation in autistic individuals presents mixed findings. Sharpley and co-workers (33) found no significant association between menarche status and depression in a sample of 53 autistic individuals aged 6–17 years. However, more concerning experiences have emerged from other studies. A mixed-method online survey on 124 participants with varying level of intellectual functioning documented frequent and severe autistic premenstrual symptoms, including increased aggression, increased repetitive movements and obsession-driven behaviours (53). Autistic individuals also exhibited a higher prevalence of behavioral issues at menses onset compared to girls with Down syndrome and cerebral palsy (38). Case reports have further highlighted severe mood and behavioral deterioration during menstruation, particularly in autistic individuals with intellectual disability (51, 55, 69). Memarian and Mehrpisheh (51) documented a case of a 12-year-old autistic individual with intellectual disability (ID) who experienced pre-menstrual “changes in mood” as well as difficulty with menstrual hygiene routines. Similarly, Skinner and co-authors (55) reported a case of an 18-year-old individual with autism and mild-moderate ID who presented with severe depressive disorder and active suicidal intent related to menstruation, along with worsening of chronic autism symptoms including obsessive-compulsive symptoms related to menstrual hygiene, episodes of agitation, aggressive outbursts, and self-mutilation. Hysterectomy was considered after an array of non-surgical options provided only limited relief. Lee (59) likewise reported two cases where self-injurious behavior occurred during premenstrual period. Carers of autistic individuals with intellectual disabilities have similarly reported behavioral and affective changes related to menstruation (50). Sensory challenges also appear significant, with many autistic participants interviewed by Grey et al. (44) describing sensory overload as a debilitating feature of menstruation. Supporting this finding, a survey by Steward et al. (35) found that autistic individuals report cyclical increase in sensory, emotional, and behavioral difficulties associated with menstrual cycle.

The prevalence of premenstrual dysphoric disorder (PMDD) in this population remains unclear, with estimates ranging from 14.5% to 92% across studies – suggesting the need for more robust, well-defined assessments (46, 57, 73). The heterogeneity of individual experiences is further underscored by findings that, in cases of precocious puberty, menstruation may be well tolerated (32) or may trigger mental health difficulties which – while not described in detail in the case series – necessitated GnRH treatment (70).

The existing research on the menopausal transition in autistic individuals remains limited, though several new studies have been published in the last few months. Two small qualitative studies by Moseley et al. (48, 68) documented that some autistic individuals experience worsening anxiety, depression, self-injurious behaviors and suicidality, and new mental health challenges, such as panic attacks, during perimenopause. These findings align with observations by Benevides and co-authors (65), who noted increased rates of anxiety, depression and sleep disturbances among menopausal autistic individuals. Supporting these quantitative qualitative insights, a quantitative study by Groenman et al. (46) demonstrated that autistic individuals had higher overall menopausal complaints, which were linked to depressive symptoms in this population. Furthermore, individuals interviewed by Moseley and co-authors (48) reported increased difficulty utilizing masking as a coping skill, worsening social communication difficulties, heightened existing sensitivities, and the emergence of new sensory sensitivities, during perimenopause. The largest international study to date, involving 508 participants, comes from Jenkins et al. (74). Many participants involved in that study indicated an increase in Autistic traits, which interacted significantly with menopausal symptoms. Additional recent studies have consistently documented increased sensory and emotional sensitivities during menopause (62–64, 66–68). These heightened sensitivities can manifest as intensified struggles with auditory and visual stimuli, challenges with transitions between tasks or settings, and difficulty in engaging with previously manageable social or professional situations (62).

### Menstrual disorders among autistic individuals

Studies consistently document higher rates of menstrual difficulties, including dysmenorrhea (42, 53, 54, 56, 58), menorrhagia (42, 55), amenorrhea (42, 56), and irregular menses (38, 54, 55, 72), among autistic individuals. Pain is a subjective and highly personal experience, shaped – among other factors – by interoceptive functioning, pain-related cognitions, (which are in turn influenced by cultural beliefs and social interpretations of noxious experiences), and social contexts (including perceived injustice or positive social interactions) (75). Experience of dysmenorrhea among autistic individuals may thus be influenced by their challenges in sensory sensitivities and in communicating their symptoms, related to verbal skills, a tendency to internalize symptoms, interoceptive difficulties, and social communication challenges (44, 50). These elements can intensify awareness of menstrual pain and create a lack of context for understanding noxious menstrual experiences (44, 50). In addition, the inability to express pain verbally may lead to failure or delay in eliciting social supports (75).

### Menstrual management in autism

The ability to manage menstrual hygiene varies widely among autistic individuals, depending on their intellectual abilities, communication skills, and motor skills (50); while some individuals manage well, others may require supports with tasks such as placing sanitary pads or cleansing. Education is a crucial aspect of menstrual management for this population, as it can effectively address the self-care difficulties reported by some autistic individuals during menstruation (41, 50, 52). Several studies have explored promising educational approaches, including “chaining” (i.e., breaking down a skill into simpler steps and having the successful acquisition of each small step reinforced) (36), using social stories (short, individualized descriptions of specific situations) (30, 71), incorporating visual methods to teach menstrual hygiene skills (40, 43), and educational interventions for health practitioners (61). Given that menstruation is a significant concern for parents of autistic individuals (34, 37, 41, 50, 51), educational strategies should involve parents where appropriate. Furthermore, the heterogeneity of individual needs should be recognized in the design and delivery of educational approaches (50) as well as the selection of sanitary products (with sensory impact being the most important feature) (39).

Eight studies indicate that autistic individuals face significant healthcare disparities related to menstruation. Many post-pubertal autistic individuals seek hormonal management of their menses, including intrauterine systems (34, 45); for example, in the cohort analysed by Fei et al. (34), 80% of post-menarchial patients desired hormonal management of menses. However, they are less likely than neurotypical individuals to visit a gynecologist or use hormonal contraception (42, 53). Similarly, autistic individuals navigating menopausal transitions report barriers to accessing healthcare and support, including medical marginalization, the extension of neuronormativity and misogyny into medical context, lack of professional awareness of the reproductive healthcare and communication needs of autistic adults, lack of resources to promote dialogue and to educate about autistic menopause (60, 64–67, 74). Encouragingly, one study found that with access to hormonal treatment, 96% of autistic participants reported satisfaction with their menstrual patterns (34). On the other hand, intense side effects of hormone-replacement therapy (HRT) were noted by some autistic menopausal participants (67).

## Discussion

Given the health risks associated with reproductive transitions (17) and the high prevalence of psychiatric comorbidities among autistic individuals, it is encouraging to note recent attempts to address the historical dearth of menstrual health research focused on autistic individuals. This shift is likely driven by changes in the research landscape, including revised gender ratios in autism diagnoses (particularly in population-based studies, where gender ratio can fall to as low as 1.8:1) (76, 77) and increased recognition of menstrual cycles as a biological variable. Initiatives such as the National Institutes of Health (NIH) “Sex as a Biological Variable” policy and NICHD's “Menstruation: Science and Society” meeting, with investigators challenged to include menstrual status in their research projects (78), has had important implications. The significance of menstrual cycle as a variable has been recognized, some grouping it together with body temperature, heart rate, respiratory rate and blood pressure as the “fifth vital sign” (79, 80). In 2023, a research paper on mental health implications of the pill pause was published in JAMA (81), and the impact of reproductive transitions on women's mental health is increasingly recognized (11).

While emerging, current evidence is based on studies involving a small number of individuals; there are only four notable exceptions; Steward et al. (35), Ames et al. (42), Pohl et al. (56), and Jenkins et al. 2024 (74). Based on the currently available data originating from three small-sample studies [*n* = 26 (57); *n* = 28 (46), and *n* = 42 (73)], there are striking differences in the reported prevalence of PMDD within the autistic population. Even though all three studies used DSM IV diagnostic criteria, there were paramount differences in the population characteristics. Obaydi and Puri (57) enrolled participants with learning disabilities residing in hospitals or care homes, whereas the other two studies recruited community-residing participants and excluded those with IQ below 80 (73) or IQ below 70 (46). Therefore, future research based on larger community populations, and employing data stratification for varying IQ levels or learning abilities, will be important to shed more light on the possible factors contributing to these marked discrepancies in PMDD prevalence.

**Figure 4:**
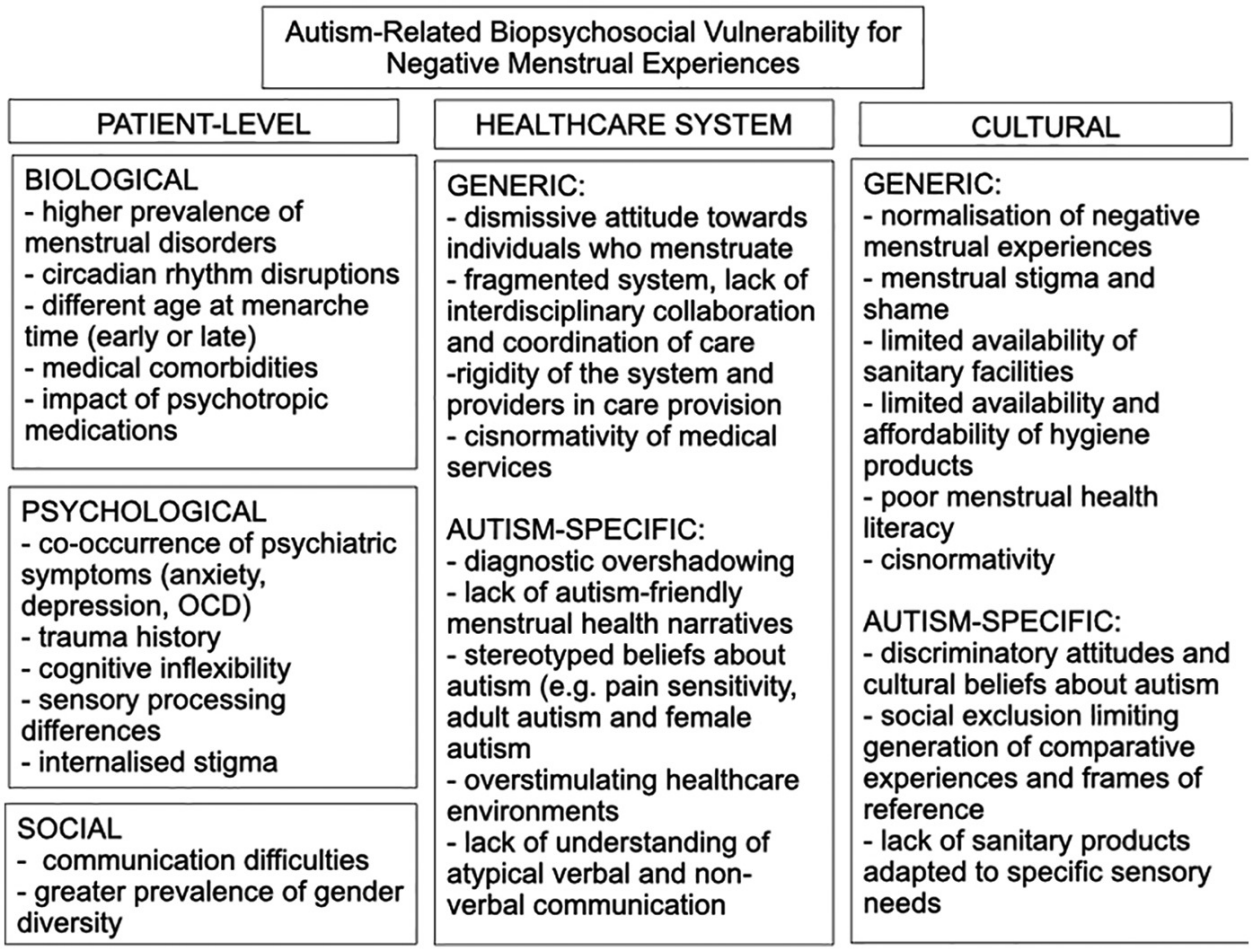
The proposed impact of ASD on the experience of menstruation at the individual, healthcare system and societal level. Autistic women have higher rate of menstrual disturbances, medical co-morbidities, circadian rhythm disruption (83, 84) - which can also impact menstrual disturbances (85), and peculiarities of exteroceptive and interoceptive sensory processing (contributing to difficulties with sight, odor and sensation of menstrual blood, and perception of menstrual cramping). The frequently observed high co-occurrence of mental health conditions in ASD could alter, in a bidirectional manner, the interoceptive functioning and menstrual symptomatology. Cognitive inflexibility may pose challenges at times of physiological variations and associated daily routine changes during menstruation. Menstrual communication, a source of reassurance and normative frames of reference in typically developing individuals (101), may be disrupted secondary to personal (communication difficulties, self-stigma), healthcare and societal factors. The diagnostic process and therapeutic engagement are likely affected by interacting with generic and autism-specific healthcare system and societal factors.

Our current knowledge on menstrual health specific to autistic individuals is still scant (31). Nevertheless, it raises significant concerns regarding potential challenges. Autistic individuals have been reported to exhibit affective and behavioral changes linked to menstruation, ranging from cyclical amplification of autism symptoms to intensification of mood symptoms and emotional dysregulation. The various elements that interact to shape the vulnerability to negative menstrual experiences within the autistic community can be examined using the bio-psycho-socio-cultural framework, which includes individual patient factors, healthcare system influences, and cultural aspects (Figure 4). Autistic females have been shown to exhibit a higher prevalence of menstrual disorders (42, 53, 54, 56), and some authors propose that hormonal differences, such as increased androgen levels, may contribute to this phenomenon (82). Circadian rhythm disturbances and sleep disorders are also commonly linked with ASD (83, 84), and it is known from studies in non-autistic individuals that most dimensions of sleep disturbances negatively impact on menstruation (85). Autistic individuals are frequently prescribed medications such as SSRIs or antipsychotics (e.g., risperidone) (86), which can sometimes lead to menstrual disorders (87, 88). Autistic individuals experience a greater exposure to adverse social life events (89, 90), including sexual victimization (91), in comparison to neurotypical individuals. In this regard, similar to neurotypical individuals, trauma and stress can lead to depressive cognitions and alterations in subjective pain experiences, potentially resulting in more severe menstrual symptomatology (92) and culminating in coping difficulty during menopause (66). Similarly, autistic individuals tend to have elevated rates of OCD (93), and it has been observed that menstruation can exacerbate OCD symptoms (94, 95), including in autistic people (53, 55). This might further contribute to the overall adverse menstrual experiences reported by some autistic individuals. Finally, stigma has been shown to affect pain perception in females and as such it may also impact experience of menstruation (96). Autistic individuals who experience menstruation belong to marginalized groups, facing public and self-stigma not only for their neurodivergence and biological sex but also due to their often-intersecting identities within the LGBTIQA+ communities (97, 98) and their encounters with mental health challenges. The convergence of these elements is expected to multiply disadvantages and stigma, in keeping with the theory of intersectionality (99, 100). Cultural factors and wider societal norms, including public and professional perceptions of autistic characteristics, representation in the media, educational resources, attitudes towards menstruation and menstrual communication (101), barriers experienced at the level of health service provision and prominent societal influences (some generic and some specific to autistic individuals), play a crucial role in shaping the experience of stigma (102) and by extension are likely to impact on menstrual experiences.

The variability in menstrual experiences among autistic individuals is notable, with some studies describing it as uncomplicated or satisfying (e.g., Fei et al. (33) or Moriuchi et al. (32). We propose that menstrual experiences of autistic individuals should be viewed through the lens of polyfinality, whereby the combination of specific autism and menstrual processes can actualize diverse psychosocial outcomes (such as employment status, social functioning and integration, psychological wellbeing). This is because menstrual experiences occur within the context of person-environment transactions, as described in our proposed biopsychosociocultural framework (Figure 4). These vulnerabilities establish probabilistic, but not deterministic, pathways to negative menstrual experiences.

The current research on menstrual experiences among autistic individuals has significant gaps. These include the influence of gender diversity (98) (e.g., experience of menstruation by transgender men or non-binary individuals), the impact of medical co-morbidities (e.g., secondary dysmenorrhea), traumatic experiences [as autistic individuals have higher rates of adverse childhood experiences (90)] and stigma, limited data on perimenopausal experiences specific to autistic individuals, the interaction between communication, alexithymia and interoception as factors affecting menstrual experiences, the need for clinical tools to assess menstrual symptoms in autistic individuals of varying communication skills, healthcare professionals' experiences in assessing and treating this population, and the development of interdisciplinary approaches (particularly combining gynecological and psychiatric expertise) to manage menstrual difficulties. Addressing these gaps would provide invaluable insights to optimize menstrual healthcare for autistic individuals.

## Limitations

As emphasized in the Results section, this is a rapidly developing field of clinical research with 15% of the included papers published this year, and our results are only up to date as of May 2025. An important paradigm is a shift towards co-produced research, where autistic individuals are included in the research process through Patient and Public Involvement or Lived Experience Advisory Panels. Although not included in this review, future reviews could include whether co-production was part of publications as another indicator of neurodiversity-affirmative processes. This study incorporated publications documenting experiences of both formally diagnosed autistic individuals and those who self-identify as autistic. This inclusive approach enabled us to capture valid lived experiences and their surrounding narratives. It acknowledges the reality that formal diagnosis is not always essential for individuals to understand and experience their autism, and reflects the practical landscape where self-identification often serves as a proxy for formal diagnosis due to widespread delayed or missed diagnoses. We recognize, however, that this methodological approach introduces sample heterogeneity, potentially limiting the generalizability of our findings.

## Conclusions

This scoping review highlights current findings and identifies gaps in the growing area of research concerning menstrual health of autistic individuals. There is a paucity of large-scale studies, and considerable variability in the methodologies used leads at times to significant discrepancies in results (e.g., on PMDD prevalence within the autistic population). Current literature emphasizes that autistic individuals face greater challenges related to mental health in connection with their menstrual experiences, including menopause, along with obstacles in accessing healthcare and support services. Interpreting these findings in the context of a broader understanding of mental health determinants and stigma affecting autistic population, we propose a bio-psycho-socio-cultural model of vulnerability to negative menstrual experiences within the autistic community, which could be used to inform future research.

## Data Availability

The original contributions presented in the study are included in the article/Supplementary Material, further inquiries can be directed to the corresponding author.
