# Supplementary material for: Autism, menstruation and mental health- a scoping review and a call to action
Source: Front Glob Womens Health. 2025 Jun 25;6:1531934. doi: 10.3389/fgwh.2025.1531934 (PMC12237902; doi:10.3389/fgwh.2025.1531934)
Supplement: Supplementary file 2 [file Table2.docx]

Supplementary Table 2. Key characteristics and findings from the studies (n=45; (17-61)) included in this scoping review. Abbreviations used: ASD, autism spectrum disorder; CP, cerebral palsy; DS, Down Syndrome; HCP, healthcare practitioners; HPA, hypothalamic-pituitary-adrenal; HRT, hormone replacement therapy; ID, intellectual disability; INT, interventional study; LNG-IUS, intrauterine system; NSAID, non-steroidal anti-inflammatory drug; OBS, observational; OCP, oral contraceptive pill; OT, occupational therapist; PMDD, premenstrual dysphoric disorder; Retro., retrospective; SI, suicidal ideation; QoL, quality of life; QRE, questionnaire.

| **Author / Year** | **Location** | **Aims** | **Design** | **Sample characteristics** | **Main findings/outcomes** | **Theme** | | |
| --- | --- | --- | --- | --- | --- | --- | --- | --- |
|  |  |  |  |  |  | **MH** | **MD** | **MM** |
| Ames et al  2024 | USA | To characterize the use of reproductive health care services in adolescent girls | Cohort Study, review of medical records | 3723 girls aged 14 – 18; 18.8% Autistic participants. | Autistic adolescent girls have a higher prevalence of menstrual disorders and are less likely to use available reproductive health care services. |  |  |  |
| Arslan et al 2024 | Turkey | To evaluate menstrual hygiene skills of Autistic adolescents, based on primary care giver reports. | Qualitative case study | 10 mothers of Autistic girls aged 11-12 | Autistic adolescent girls displayed sub-optimal menstrual hygiene skills and exhibited increased aggressive and obsessional behaviours during menstruation, leading to increased strain in mother- daughter dyad. |  |  |  |
| Benevides et al 2024 | USA | To investigate experience, and factors associated with symptomatic menopause | Retro. cohort study; healthcare claim record analysis | 26,904 Autistic respondents aged 35-70; 68% of the cohort had ID | Individuals with co- occurring intellectual disability, were less likely to report to symptomatic menopause, highlighting the need for adaptive psychosocial scaffolding.  Increased rates of anxiety, depressive disorders, altered sensation and sleep disturbance during menopause. |  |  |  |
| Boxerman et al 2025 | USA | Efficacy of ‘period kits’ | Mixed methods | 9 child/adult pairs | Limited baseline knowledge about menstruation was amenable to change with the studied intervention |  |  |  |
| Brady et al 2024 | Canada and UK | To explore experience of menopause and access to services | Focus group and semi-structured interviews; qualitative study | 24 autistic participants, aged 40-71 years. | Themes generated included: heightened mental health struggles, emotional and sensory sensitivities; barriers to support, complexity of symptoms, the importance of education and converging psychosocial adversity during menopause |  |  |  |
| Burke et al 2010 | USA | Identification of presenting complaints among patients presenting to a specialized gyneacological clinic | Retro.  review of medical records | Mean age 15 +/- 3.5 years | Girls with autism were significantly more likely to present with behavioural issues than girls with DS or CP. Management of behavioural issues related to the onset of periods included NSAIDs, OCP and education. Other frequent complaints included irregular bleeding. |  |  |  |
| Charlton et al 2025 | UK | To explore self reported menopausal symptoms | Cross-sectional online survey; quantitative | 342 respondents, 70% autistic, aged 40-86 | Higher rates of burdensome psychological and somatic, but not vasomotor, menopausal symptoms reported by autistic respondents |  |  |  |
| Chen et al 2014 | Taiwan | N/A | Case report | 13 y/o Autistic girl with ID and dysmorphic phenotypic features | Poor menstrual hygiene skills and genetic anomalies noted. |  |  |  |
| Cridland et al 2014 | Australia | Characterisation of experiences of adolescent girls with ASD | Semi-structed interviews; qualitative | 3 mother-daughter dyads and 2 mothers | Mothers encountered significant challenges when their daughters experienced puberty. Adolescent girls experienced difficulties adjusting to the increased demands of adolescent hygiene and sex-specific puberty issues |  |  |  |
| Cummins et al 2020 | UK | To investigate experiences of puberty for minimally verbal autistic girls | Semi-structured interviews; qualitative study | Ten parents of 9 autistic girls, average age 14years 8 months | Heterogeneity in experiences of puberty, in needs and in management of menstrual hygiene. Changes in behaviour were noted by many in relation to menstruation. Concern about inability to express pain in daughters / clients during menstruation. Respondents emphasized importance of teaching menstrual care skills using simple steps and a range of strategies. |  |  |  |
| Cusano et al 2025 | USA | To understand experience of menopausal transition among autistic individuals | Video-phone interviews; Qualitative study | 21 autistic adults with experience of perimenopause | Respondents reported increased sensory sensitivities which severely impacted their QoL, and insufficient support from healthcare providers |  |  |  |
| De Visser et al 2025 | UK | To explore autistic people’s experiences in reproductive and sexual health | Mixed methods - Online surveys | 136 autistic adults; mean age 40.2; 92% female respondents | Participants were more comfortable discussing physical than menstrual- or menopause-related issues with healthcare providers, and did not feel that HCPs had awareness of how autism may affect reproductive health |  |  |  |
| Fei et al 2021 | USA | Characterisation of patients presenting for anticipatory guidance and menstrual management | Retro.  cohort study; quantitative | 478 girls with special needs; 40% Autistic; average age of 10.7 | Patients with ASD more likely to present for a premenarchial visit – 30% of patients  Anticipation of puberty was a cause of anxiety in families and patients with ASD  80% patients desired hormonal management of menses |  |  |  |
| Ferrara et al 2024 | Italy | Thematic analysis of menstruation in autism | Scoping review | 5 studies | Lack of in-depth understanding of the topic in current literature  Deterioration in sensorial perception, anxiety and depressive symptoms during menstruation |  |  |  |
| Gray et al  2023 | UK | To explore the experiences of period pain and treatment uptake. | Qualitative interview study | 37 women; 45.9% of participants were Autistic; mean age of 27.7 years. | Autistic participants identified heightened sensory challenges and emotional dysregulation during menstruation, difference in menstrual pain expression, and lack of framework of reference for period pain and menstruation due to social isolation. |  |  |  |
| Groenman et al 2022 | Netherlands | To determine prevalence of PMDD and menopausal symptoms among autistic and typically-developing individuals | Cross-sectional; quantitative | 28 autistic women aged 49.8 for PMDD, 30 autistic women aged 58.5 for menopause  Total of 77 comparator participants | There was no increase in prevalence of PMDD in autistic women  Autistic women experienced higher total menopausal complaints – increased irritation, hyperactivity/impulsivity, and depression |  |  |  |
| Hamilton et al 2011 | USA | To evaluate experience of menstruation of Autistic girls | Online focus group; mixed methods | 124 primary care givers of Autistic girls aged 10-25 years. 5 self-responders. | Increased rate of dysmenorrhea and menstruation-related mood changes in Autistic girls. |  |  |  |
| Ingudomnukul et al 2007 | USA | To evaluate whether autistic women have increased rate of testosterone-mediated conditions | Cross-sectional QRE; quantitative | 54 autistic women, 74 mothers of autistic children, 183 mothers of typically-developing children | Autistic women have higher rate of menstrual cycle irregularities and dysmenorrhea |  |  |  |
| Jenkins at al 2024 | UK | To explore the menopause symptoms and relationship between menopause difficulties and autism awareness | Mixed methods; international survey | 508 Autistic respondents; average age 45.9 | Most often reported symptoms were memory/concentration changes, mental health changes, sleep disturbances and vasomotor symptoms; 54% entered menopause unaware of their autism; 52.3% reported symptoms as more intense than expected, and 83.5% lacked knowledge on autism-related aspects of menopause. Main themes were the gaps in knowledge and representation, struggles with well-being (exacerbated autistic symptoms, emotional volatility, and suicidality), barriers to support and healthcare (medical marginalisation, misogyny, communication challenges), experience of social isolation and aspects of greater well-being during and after menopause |  |  |  |
| Karavidas & de Visser, 2022 | UK | To examine how autistic people conceptualise and negotiate menopause | Qualitative semi-structured interviews | 7 autistic individuals, aged 39-63 | Respondents described difficulties in identifying and understanding physical and emotional alterations of menopause and heightened emotional and sensory sensitivities. Cessation of menstruation was considered a relief. 4 participants reported intense side effects of HRT |  |  |  |
| Kaydirak et al 2023 | Turkey | To evaluate the response to menstrual hygiene skills training | INT study | 15 Autistic girls aged 9-18 years. | Improved menstrual hygiene skills after training. |  |  |  |
| Klett et al 2012 | USA | To evaluate the effectiveness of parent implemented Social Story intervention in menstrual hygiene skills training | INT study | Parents of 3 autistic girls | Parents reported high satisfaction with intervention procedures and outcomes. |  |  |  |
| Kumar and Shweta, 2024 | India | To evaluate the effect of video modelling and simulation to improve menstrual hygiene skills in Autistic adolescents. | INT Study | 49 autistic girls, aged 12 to 18 years | Improvement in menstrual hygiene was noted for both video modelling and conventional OT intervention, but effectiveness was higher in the video modelling approach |  |  |  |
| Kyrkou 2005 | New Zealand and Australia | To explore experience of dysmenorrhea and Premenstrual syndrome | Cross-sectional, Qualitative study | 24 parents of women with Down syndrome or Autistic women with ID | Women with DS were more often able to state that they had pain or point to the location of the pain than women with Autism. Additionally, women with DS or ASD appeared to have a higher rate of period pain than women in the general population, but the presence of pain more often had to be deduced from behavioural changes. |  |  |  |
| Larson et al 2021 | USA | To identify puberty-related interventions used by OTs working with Autistic adolescents | Cross-sectional survey; quantitative study | 71 OT practitioners, with an average of 16yrs experience | Few (<26%) OT practitioners addressed menstruation challenges |  |  |  |
| Lee  2004 | USA | N/A | Case report | 2 Autistic girls | Self-injurious behaviour coinciding with PMDD |  |  |  |
| Lever et al  2016 | Netherlands | Comparing the co-occurrence of psychiatric symptoms in typically developing and autistic adults | OBS; quantitative study | 172 Autistic adults of which 56 were female and 172 adults without ASD of which 75 were female | The lifetime prevalence of PMDD was 20.9% in the Autistic female participants vs 2.7% in comparator group |  |  |  |
| Lundy et al 2025 | USA | To characterize menstrual product preferences for young autistic people and caregivers | Cross-sectional qualitative study | 99 caregiver- youth dyads | Autistic youth’s preference was for period underwear, while for caregivers the preference was based on sensory impact |  |  |  |
| Lutz et al 2023 | USA | To analyse the use and safety of levonorgestrel-releasing IUS | Retro. review of medical records | 1560 female adolescents with developmental delays; aged 10-25 | LNG-IUS insertion was commonly performed in Autistic patients |  |  |  |
| McMahon et al 2025 | USA | Impact of educational intervention for the care of autistic adolescents | INT study | Healthcare personnel – family nurse practitioner | Increase in self-perceived knowledge, attitudes and intent to change practice among family nurse practitioners following educational intervention that involved skills in menstruation management |  |  |  |
| Memarian et al 2015 | Iran | N/A | Case report | 12yo Autistic girl with ID | Poor menstrual hygiene skills and menstruation-related mood changes noted, leading to referral for hysterectomy. Authors advocate for culturally-informed and individual management of menstrual difficulties. |  |  |  |
| Moriuchi et al 2023 | Japan | Clinical case report | Case report | 2 girls with ASD and CPP, ages 7 and 9 years | Heterogeneity in psychosocial burden of menarche |  |  |  |
| Moseley et al 2020 | UK | To investigate the state of knowledge about the menopause in autistic women | Online focus group; qualitative study | 7 Autistic women; average age 54.8 | Menopause may magnify and even generate new difficulties with sensory sensitivities, communication, emotion regulation and everyday living |  |  |  |
| Moseley et al 2021 | UK | To explore awareness of menopause and menopausal experiences | Semi-structured interviews; qualitative study | 17 Autistic individuals; aged 41-66 | Worsening of pre-existing anxiety and depression during menopause, in some cases with increased self-injury, SI or attempts; increased emotional lability, and meltdowns; lower ability to socialize, greater difficulties with non-verbal and verbal communication, heightened sensory sensitivities, Few respondents were in receipt of HRT |  |  |  |
| Obaydi and Puri, 2008 | UK | To determine the prevalence of late luteal phase dysphoric disorder | Prospective observer-rated study; quantitative | 26 Autistic women with ID and 36 non-autistic women with ID | Prevalence of late luteal phase dysphoric disorder was 92% in the autism group vs 11% in the control group |  |  |  |
| Piper & Charlton, 2025 | UK | To explore experiences of menopause | Semi-structured interviews; Qualitative study | 15 Autistic (aged 44-66) and 14 non-autistic adults (aged 45- 84) | Themes common to both groups: lack of education, the importance of social supports, and negative psychological changes during menopause. Autistic respondents identified need for scientific information, challenges associated with change, unpredictability and interoceptive difficulties, and barriers in accessing medical help |  |  |  |
| Pohl et al 2014 | UK | To assess the frequency of steroid-related conditions and symptoms | Cross-sectional online survey; quantitative study | 415 Autistic women (mean age 36) and 415 controls ( mean age 39) | Autistic women had a higher frequency of amenorrhea and, dysmenorrhea |  |  |  |
| Sharpley et al 2021 | Australia | Identification of correlates of depression in young autistic females | Cross-sectional study; quantitative | 53 Autistic girls, aged 6-17 | Self-reported depressive symptomatology was associated with HPA-axis responses but not with menarche status |  |  |  |
| Simantov et al 2021 | UK | To explore association between autism/ autistic traits and sex- steroid disorders in adult women | Case-cohort; quantitative study | 1230 women aged 15-77 years. 29.3% of respondents were women with autism/ autistic traits. | Higher rates of excessive menstrual symptoms and prolonged menstrual length were associated with individuals with a diagnosis of autism or autistic traits. |  |  |  |
| Skinner et al 2005 | Australia | N/A | Case report | 18yo Autistic woman with ID | Management of severe affective disorder related to menstruation, including use of subdermal etonogestrel implant and antidepressants, and ultimately a plan for hysterectomy |  |  |  |
| Steward et al 2018 | UK | Investigation of the experiences of post-menarcheal autistic and nonautistic respondents | Cross-sectional; qualitative study | 237 women, 51.9% Autistic, aged 16-60 | Exaggerated sensory issues and intensified executive and emotional problems in relation to menstruation |  |  |  |
| Veazey et al 2015 | USA | Teaching hygiene skills | INT study | 2 Autistic girls with ID, aged 9yo and 11yo | Example of chaining procedures to target feminine hygiene in individuals with ASD and ID |  |  |  |
| Vijapura et al 2014 | USA | N/A | Case report | 16yo Autistic girl with ID | Management of severe affective disorder related to menstruation |  |  |  |
| Wild & Tobe, 2019 | USAS | N/A | Case report | 17yo Autistic girl | Since menarche, the frequency of pica behaviours increased before periods, returning to baseline at the end of menstruation. OCP was successful in reducing pica behaviour |  |  |  |
| Yoshirmura et al 2004 | Japan | N/A | Case report | 3 Autistic girls with precocious puberty, aged 6-9 years | All three patients experienced psychological burden secondary to menstruation, necessitating GnRH treatment to stop menstruation |  |  |  |
